# Duodenal Perforation in a Neonate: An Unusual Presentation and Analysis of the Cause

**Published:** 2015-04-01

**Authors:** Yasser Al Omran, Mohammed Omer Anwar, Saeed Al-Hindi

**Affiliations:** 1 Barts and the London School of Medicine and Dentistry, Garrod Building, Turner Street, Whitechapel, London, E1 2AD, United Kingdom.; 2 Salmaniya Medical Complex, Manama, Bahrain

**Keywords:** Duodenal perforation, Neonate

## Abstract

Duodenal perforation in neonates is a rare surgical emergency. In the cases reported, most perforations are localised to the anterior duodenum and a few at posterior aspect. We present a case of duodenal perforation in the second part of the duodenum in a 26-day-old healthy male neonate.

## INTRODUCTION

Gastrointestinal perforations in neonates are associated with a high mortality. [1] Neonatal duodenal perforations are rare as there are only a few cases have been reported in the English literature. [1-4] Peptic ulcer disease, enteral tube feeding, and the use of ventilators are implicated in its aetiology. In some of the cases no cause could be found and these are reported as “spontaneous” to describe a perforation without an obvious cause. [1,2] However, it has been suggested that when a perforation occurs in a neonate, a contributing cause should be sought. [5] 

## CASE REPORT

A boy of Indian origin was born at 36 weeks gestation by emergency caesarean section weighing 2985g. His Apgar score at 1 minute was 6, and at 5 minutes had increased to 8. His health status was unremarkable from Day 1 onwards until he presented to hospital on the 23rd post-natal day with a 24-hour history of fever, persistent dry cough, wheezing and poor feeding. On examination, the patient was tachypnoeic, pyrexical (38.9°C) and widespread fine inspiratory crackles were auscultated throughout the lung fields. His chest x-ray was normal. A Virology testing of a nasopharyngeal swab suggested infection with respiratory syncytial virus (RSV) and a diagnosis of bronchiolitis was made. The patient had a nasogastric tube (NG) inserted for feeding and was admitted into the paediatric intensive care unit where he was managed with oxygen driven nebulised salbutamol and intravenous crystalloid hydration for 2 days before being transferred to the general paediatric ward when his condition settled. 

Whilst on the ward, the patient developed abdominal distension and bilious vomiting. His NG tube remained in-situ at the time and wasn’t removed. On examination, his abdomen was soft with ill-defined peri-umbilical tenderness. Bowel sounds were absent. Blood tests were notable for a raised white blood cell (WBC) count of 13.9 x 109/L and a C - reactive protein (CRP) of 60 mg/L. A subsequent supine chest and abdominal X-ray of the abdomen revealed a large pneumoperitoneum.

During laparotomy bile was noticed in the abdomen and after examining the stomach, small and large bowel, it was decided that the duodenum should also be checked from anterior and posterior surfaces. Peritoneal toileting with normal saline and a complete kocherization (mobilisation) of the duodenum was undertaken. A biliary leak was noted due to a 1cm perforation on the posterior wall of the second part of the duodenum, just distal to the major duodenal papilla. As the visualised mucosa around the perforation appeared normal, and to avoid potential damage of ampulla of vater or stricture formation after healing; a biopsy wasn’t undertaken and the perforation was closed in a single layer of interrupted 5-0 silk sutures in a transverse manner and an onlay pedicled omentoplasty was used to cover the defect. A trans-anastomotic tube was inserted and pushed distally for early postoperative feeding. 

The postoperative course was uneventful. The patient’s bronchiolitis soon resolved and a dye study done on the 11th postoperative day showed an intact duodenum. At this point, the feeding tube was removed. Presently the child is 3 months of age and is thriving well.

## DISCUSSION

Duodenal perforations in neonates are rare and only a few cases are reported in the English literature. [1-4] Most of the perforations are on the anterior aspect of the duodenum. We present a case of perforation at the posterior aspect of the second part of the duodenum. To the best of our knowledge, a neonatal posterior duodenum perforation in the second part of the duodenum has only been reported once before in the English literature. [3] The perforation of duodenum at this location requires special attention, as this is the point at which the pancreatic and common bile ducts open through the major duodenal papilla. Damage to these structures would have caused a far more serious clinical presentation. Additionally, posterior duodenal perforations may be difficult to identify and may be missed altogether, as has been previously reported. [5,6] Thus, mobilisation of the duodenum is necessary whenever there is a suspected perforation. Furthermore, a thorough examination of the duodenum should be initiated to rule out concomitant anterior and duodenal perforations. [6,7] 

The causes of duodenal perforation in neonates have not been clearly identified. Nearly half of the reported duodenal perforations in neonates have been due to a perforated duodenal ulcer. With regards to our case, it is unlikely that the cause for duodenal perforation would be attributed to a possible secondary ulcer (e.g. due to the stress caused by bronchiolitis). This is because there have been less than 30 such ulcer-related causes for perforation in the posterior aspect of the duodenum reported in both the paediatric and adult population. [8] Moreover, as local inflammatory reactions and fibrosis of the retroperitoneal tissue tends to seal off these perforations, in those reports of posterior duodenal perforation, peritonism was not seen. [8]

The insertion of feeding tubes has also been reported to cause duodenal perforation. [9] It appeared from chest and abdominal radiography, that the distal tip of the NG tube was located in the lower body of the stomach, with nothing to suggest penetration into the lumen of the duodenum. Therefore, it seems unlikely for this to have been the cause of the perforation. Finally, it has been reported that out of 20 neonates with gastrointestinal perforations, 15 had received mechanical ventilation via nasal cannulae or facemasks. [10] The proposed aetiology was that of initial ischaemic injury caused by asphyxia and increased intraluminal pressure from the ventilation leading to the perforation. Although these perforations were limited to the first week of life, the prolonged use of oxygen driven nebulised salbutamol and presentation of dyspnoea may have been a contributing cause to the duodenal perforation in our patient. Even though a definitive cause is difficult to determine in this case, duodenal perforations are associated with a high mortality. [1-3] Therefore, greater awareness of the causes of duodenal perforations is of vast importance; clinicians should be aware of these causes to allow for earlier identification of perforation, and to facilitate better patient outcomes.

## Footnotes

**Source of Support:** Nil

**Conflict of Interest:** None

